# Prolonged Remission in Metastatic Ano‐Rectal Malignant Melanoma With Single Agent Temozolomide

**DOI:** 10.1002/cnr2.70121

**Published:** 2025-01-14

**Authors:** Anusha Mruthyunjaya Swamy, Deepak Sundriyal, Mayank Kapoor, Mridul Khanna, Ravi Hari Phulware, Kranthi Kumar Jandrasupalli, Ujjawal Shriwastav, Amit Sehrawat

**Affiliations:** ^1^ Department of Medical Oncology, Hematology All India Institute of Medical Sciences Rishikesh Uttarakhand India; ^2^ Department of Pathology and Laboratory Medicine All India Institute of Medical Sciences Rishikesh Uttarakhand India; ^3^ All India Institute of Medical Sciences Rishikesh Uttarakhand India; ^4^ Chitwan Medical College Bharatpur Nepal; ^5^ Tribhuvan University Kirtipur Nepal

**Keywords:** ano‐rectal melanoma, chemotherapy, complete metabolic response, temozolomide

## Abstract

**Introduction:**

With the use of immune checkpoint inhibitors (ICIs) and targeted therapies, the clinical outcomes of metastatic melanoma have drastically improved. The current scenario has reduced the use of chemotherapy as a first‐line treatment. We report an interesting case of a patient with stage IV ano‐rectal canal malignant melanoma with an exceptional response to single‐agent temozolomide.

**Case Report:**

We diagnosed a 55‐year‐old female with stage IV anorectal melanoma, BRAF V600 mutation negative. Owing to her poor performance status (PS) and non‐affordability of immunotherapy, after informed decision‐making, she was started on single agent, temozolomide. She achieved a complete metabolic response and sustained it for 3 years and continues to do so with the first‐line single‐agent temozolomide.

**Conclusion:**

In a resource‐limited setting, where access to ICIs and targeted therapies is not feasible, and in patients who fail to tolerate these therapies, oral chemotherapy continues to remain effective and is worth trying in patients with poor PS.

## 1 Introduction

Metastatic melanoma has historically been a very challenging entity for clinicians due to limited effective therapeutic options and the disease being chemotherapy‐resistant. Before discovering Immune Checkpoint Inhibitors (ICIs) in 2011, the median survival with dacarbazine as single agent chemotherapy ranged from 8 to 12 months. With ICIs, the median overall survival has improved to at least 24 months [1]. However, many patients fail to respond, have poor tolerance to ICIs, or develop secondary resistance, and the treatment of such patients, especially those lacking BRAF V600 mutations, becomes difficult as the standard treatment options available are suboptimal [2]. Though chemotherapy previously had minimal anti‐tumor activity with low response rates and short duration of disease control, recent data suggest that its action on tumor microenvironment might help boost tumor‐directed immune responses [3]. Temozolomide is a potentially attractive chemotherapeutic agent in such patients because of its oral administration route, easy availability, low cost, and ability to cross the blood–brain barrier, a common site of metastases in melanoma [4].

While there is limited evidence for combining chemotherapy and immunotherapy, the objective response rates to single‐agent temozolomide are still dismally low at approximately 13% [5]. However, in a resource‐limited clinical setting like in India, and in a patient with a poor performance status (PS), low‐cost oral therapy is worth trying. Here, we report an interesting case of a patient with stage IV, poor PS, ano‐rectal canal malignant melanoma with an exceptional response to single agent temozolomide, with a prolonged Complete Metabolic Response (CMR) for more than 3 years [4]. The perplexity of the case lies in the fact that while most of these patients (resource‐limited, poor PS) are destined for best supportive/hospice care, few of them may enjoy a quality and prolongation of life with the oral low‐cost therapy without much side effects.

## 2 Case Report

**FIGURE 1:**
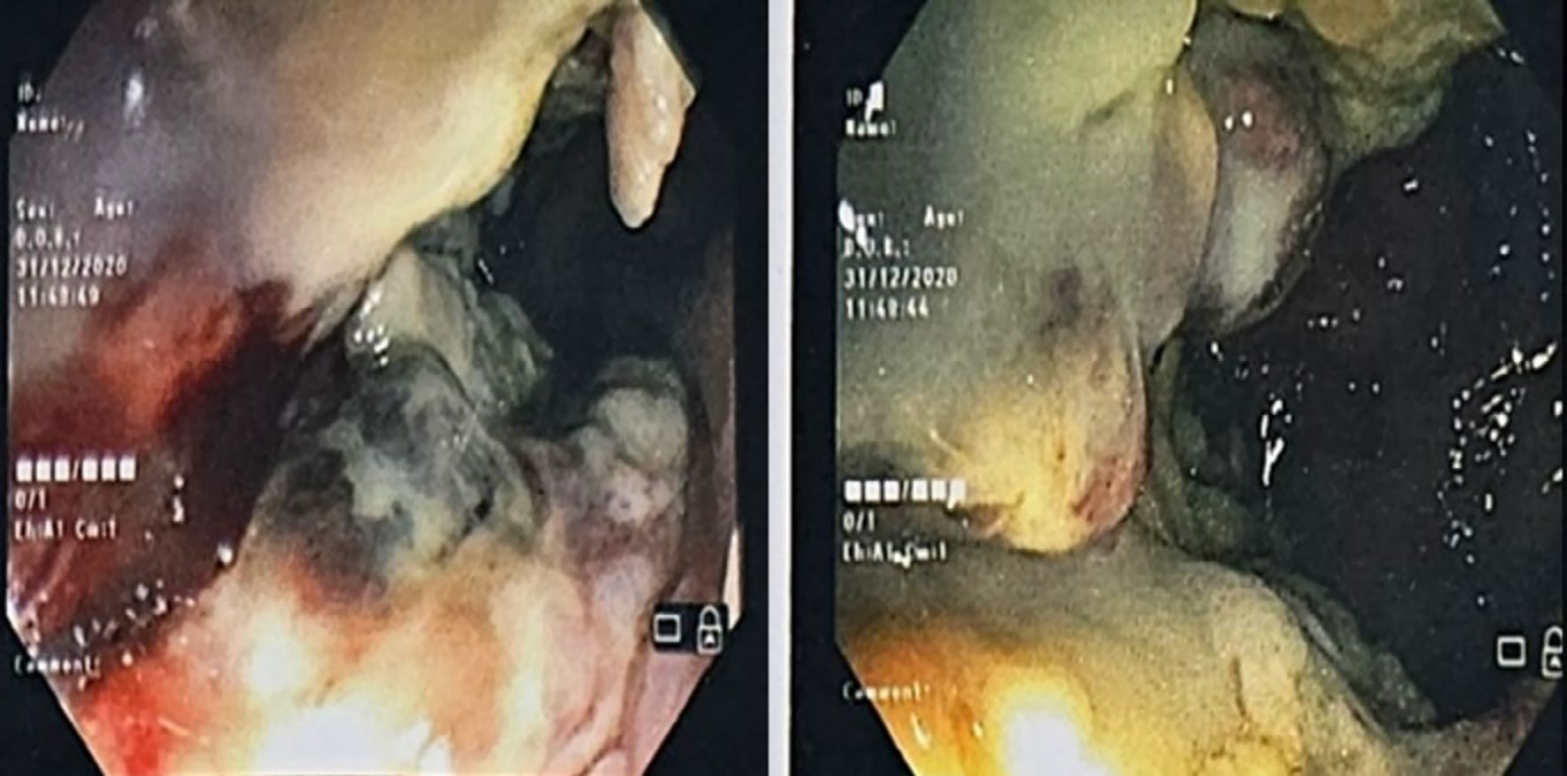
Colonoscopic findings an ulceroproliferative growth visualized in the anal canal, extending upto the rectum.

**FIGURE 2:**
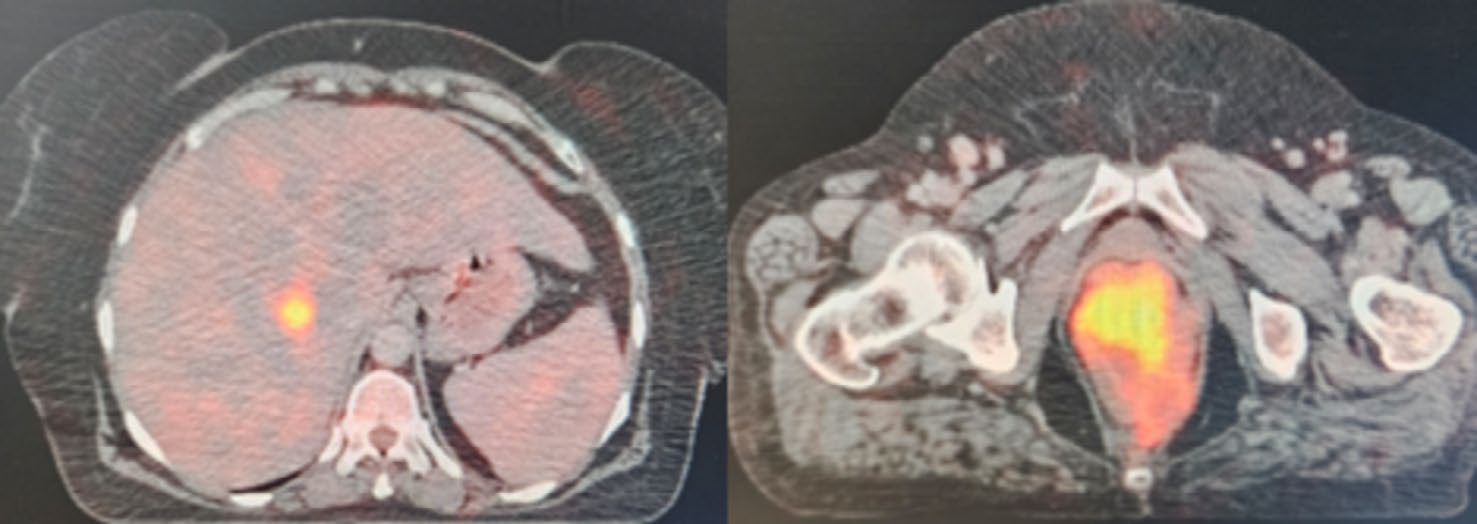
Liver metastases and the ano‐rectal growth as visualized in the baseline whole body FDG18 Positron Emission Tomography CT (PET CT).

**FIGURE 3:**
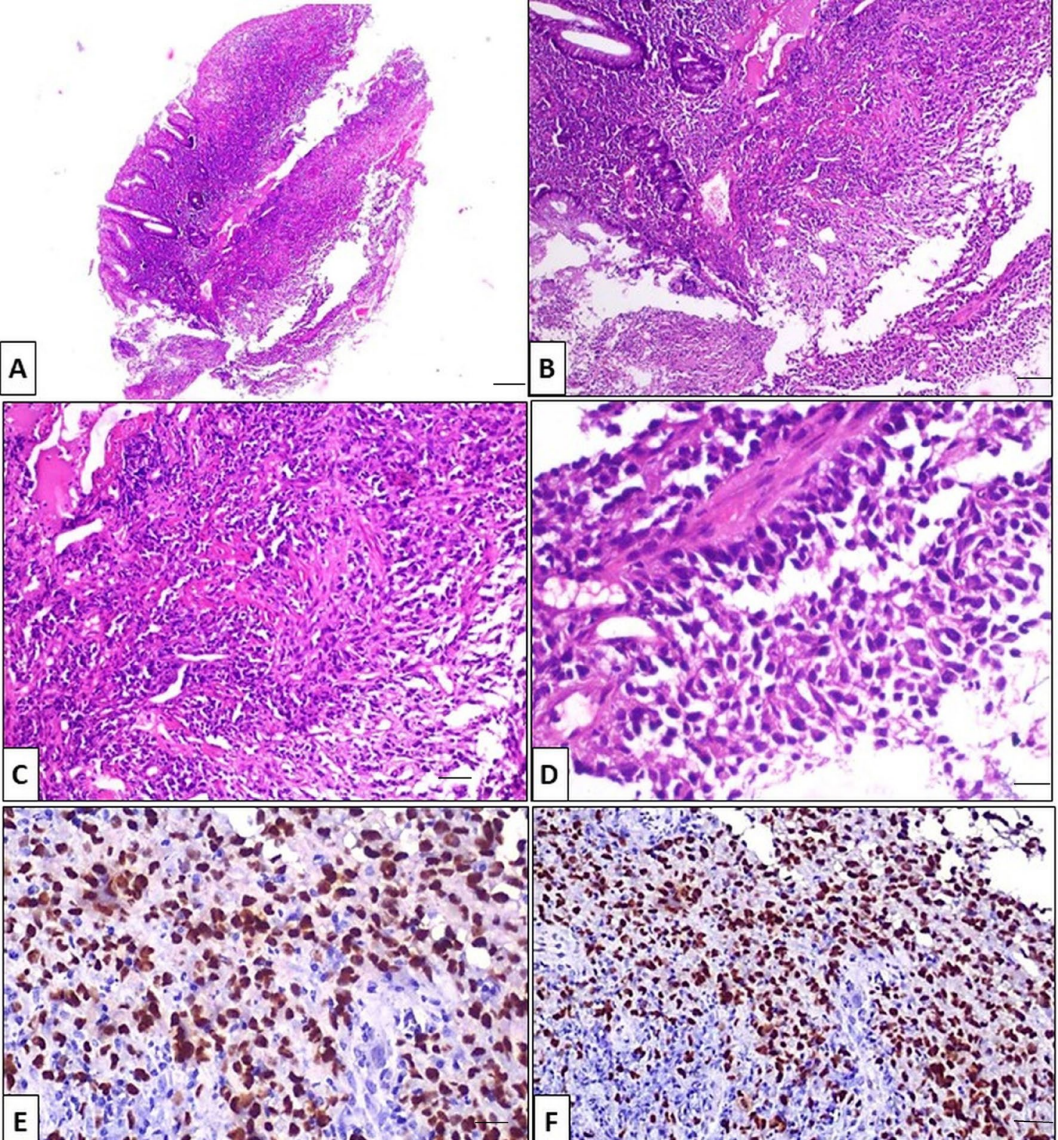
(A) Haematoxylin and eosin (H&E) stained section from the anal canal growth shows few colonic mucosal glands with dense inflammation, Scale bar = 4 μm, (magnification ×40). (B) and (C) Higher magnification shows loss of crypts along with atypical cells arranged singly and in groups within the inflammatory cells (scale bar = 10 μm, magnification ×100; scale bar = 20 μm, magnification ×200, respectively). (D) These atypical cells show increase nuclear cytoplasmic ratio, hyperchromatic nucleous with prominent nucleoli (scale bar = 40 μm, magnification ×400). (E) The tumor cells are positive for HMB45 (scale bar = 20 μm, magnification ×200). (F) The tumor cells are positive for Melan A (scale bar = 20 μm, magnification ×200).

A 55‐year‐old female patient with a history of type II diabetes presented to All India Institute of Medical Sciences, Rishikesh, India, in January 2021, with a 4‐month history of hematochezia, pain during defecation, loss of appetite, and weight (10 kgs in the preceding 3 months). She was chair‐bound with an Eastern Cooperative Oncology Group‐ PS (ECOG‐PS) of 4. A general physical examination revealed cachexia and pallor. On digital rectal examination, she was found to have a circumferential, ulceroproliferative growth involving the anal verge, extending up to 5 cm anteriorly. The rest of the systemic examination was regular. Blood investigations showed anemia (Hemoglobin‐8.2 g/dL), along with a serum CEA of 2.48 ng/mL (Normal value: < 5 ng/mL) with a normal total leucocyte and platelet count. Renal and liver function tests were within normal limits. On colonoscopy, the ulceroproliferative growth was visualized in the anal canal, extending up to the rectum, and multiple biopsies were taken (Figure 1). Whole‐body FDG PET CT showed FDG avid circumferential wall thickening in the distal rectum and anal canal (SUV max‐18.5), FDG avid perirectal bilateral external iliac, bilateral inguinal lymph nodes (largest measuring 1.6 × 1.1 cm and SUV max‐2.7) with FDG avid hypodense lesions in segment V and VIII of the liver (SUV max 10.3) (Figure 2). Histopathological examination and immunohistochemistry confirmed the diagnosis of malignant melanoma, BRAF V600 negative (Figure 3). Thus, she was diagnosed to have stage IV (cT4N1aM1) ano‐rectal malignant melanoma.

**FIGURE 4:**
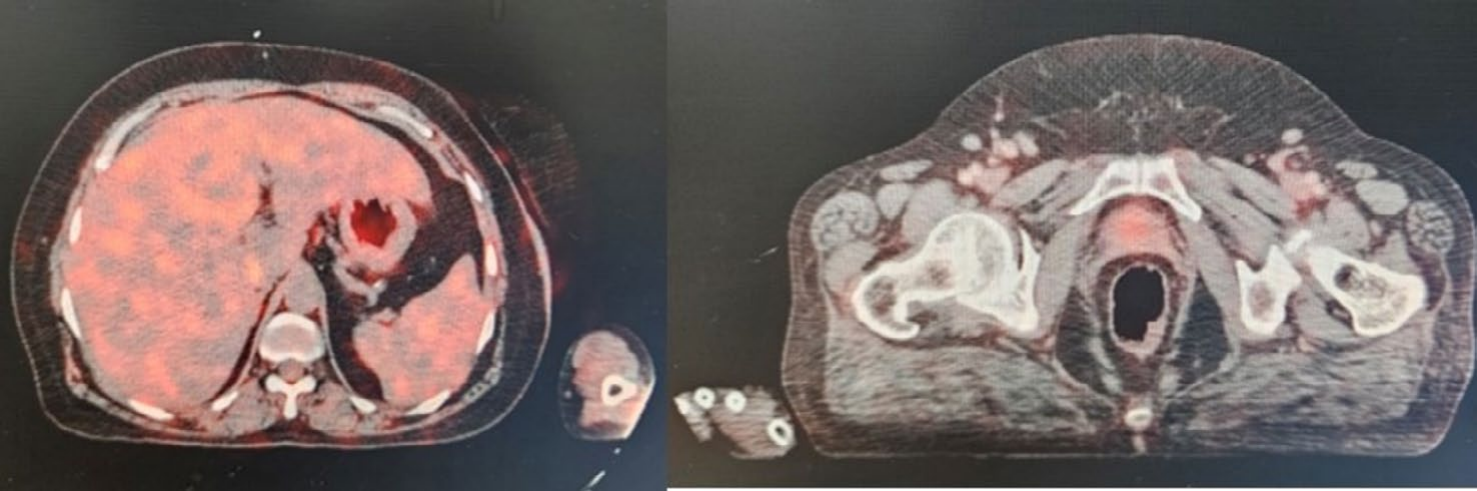
FDG 18 PET CT images demonstrating persistent complete metabolic remission (at the end of 3 years of follow‐up).

Her case was discussed in the multi‐disciplinary tumor board meeting. Owing to her poor PS of 4 and unaffordability of ICIs, after informed decision‐making, she was started on single agent Temozolomide 200 mg/m^2^ (Day 1–5, q28 days) in April 2021 along with Pneumocystis jiroveci prophylaxis as per guidelines. The patient demonstrated clinical response, and her ECOG PS improved to 2 by the third cycle. By the fifth cycle, her PS further improved to 0. CMR was first noted after the seventh cycle of temozolomide in November 2021. Grade 2 nausea and grade 1 transaminitis were noted on one occasion, requiring anti‐emetic prophylaxis. Notably, the patient did not experience any grade 3 or 4 toxicities requiring dose modification/discontinuation of the drug. Furthermore, serial images have demonstrated a persistent CMR, with the response for more than 3 years (last followed up in July, 2024) of treatment and counting (Figure 4).

## 3 Discussion

Modern treatment options, including immune checkpoint inhibitors and targeted therapy, have improved the outcomes in patients with metastatic melanoma, making the use of chemotherapy redundant. However, in countries like India, with limited resources, in patients who progress on ICIs, and in those not tolerating ICIs, chemotherapy is the backbone of treatment. Dacarbazine (DTIC), previously considered the standard of care, was the first cytotoxic chemotherapeutic agent approved by the FDA (in 1975) for metastatic melanoma. The overall response rate (ORR) with dacarbazine varies from 9% to 29% in the first‐line setting; however, durable complete responses are rare (< 2%). Other chemotherapy agents routinely used include taxanes, platinum compounds, and fotemustine. The ORRs, as reported across multiple studies with paclitaxel (0%–36%), docetaxel (5.7%–17%), Nab‐Paclitaxel (12%–15%), Cisplatin (16.3%–19.8%), and carboplatin (19.2%) are low and sustained complete response is rare [5]. Temozolomide, an oral alkylating agent with structural and functional similarity to dacarbazine, is another attractive treatment option due to its lower cost, excellent oral bioavailability of approximately 100%, and Central Nervous System (CNS) penetration [6]. The activity of Temozolomide in metastatic melanoma has been established by several phase I/II studies and confirmed by a phase III trial, which demonstrated similar ORRs between dacarbazine and temozolomide with a numerically higher progression‐free survival (PFS) and overall survival (OS) with temozolomide [7].

Chemotherapy appears to play a role by enhancing the tumor microenvironment through its action on immune cells, cytokines, clusters of differentiation, cell adhesion molecules, and major histocompatibility complexes, resulting in the alteration of the immunosuppressive environment in the tumor [3]. Furthermore, post temozolomide administration, an increase in CD8+ T cells and a decrease in CD4+ and regulatory T cells has been noted. Dacarbazine also increases NKGD2 ligands in melanoma cells, activating NK cells and secretion of interferon‐gamma. All these mechanisms and the alkylating effects of temozolomide and dacarbazine contribute to their efficacy in melanoma [8].

With extensive literature search, we found no other case reported with such durable CMR with single agent temozolomide as a first‐line agent in a metastatic setting. Maggie et al. reported a patient attaining CMR after four cycles of temozolomide after the failure of five prior lines of treatment. The duration of CMR sustained is ambiguous [1]. In another case series by Goodman et al., among metastatic melanoma patients receiving Nivolumab and temozolomide combination regimens, one patient had a sustained ongoing CMR of greater than 6 months [2]. The efficacy of temozolomide in achieving ORR and improving PFS and OS by a few months in patients who progress or those with poor tolerance to ICIs has been confirmed by several authors [3, 9, 10]. The response to temozolomide after prior exposure to ICIs has been explained by the fact that ICIs cause activation of T cells, further improving chemosensitization [11]. Our patient, however, was ICI naive. What makes our case unique is the prolonged duration of response of approximately 3 years, which continues in a first‐ line setting.

Various factors have been cited for resistance to temozolomide therapy; however, elevated O6—mrthylguanine‐DNA methyltransferase (MGMT) levels have been associated with temozolomide resistance and its expression could potentially be used to tailor therapy [5]. Although, we were not able to do a molecular analysis of the biopsy specimen in our case, the reason for the sustained response could be a low level of MGMT activity and the ability of the drug to cross the blood–brain barrier and prevent the CNS spread of disease.

Moreover, most patients with a poor ECOG‐PS of 4 are eligible only for the best supportive care. However, we considered her for a trial of oral temozolomide therapy with positive results. The inherent tumor biology appears to determine the response to chemotherapy in ICI‐naive melanoma patients.

## 4 Conclusion

Though ICIs and targeted therapies are the cornerstones of treatment in metastatic melanoma, our case report demonstrates that a sustained CMR can be achieved in ICI‐naive patients with single‐agent temozolomide, even in stage IV disease. This case highlights that a small proportion of selected poor PS patients who are otherwise bed/chair‐bound due to the disease burden may indeed benefit from a trial of an oral agent.

## Author Contributions

**Anusha Mruthyunjaya Swamy:** conceptualization, writing original draft, editing. **Deepak Sundriyal:** diagnosis and treatment, writing original draft, reviewing. **Mayank Kapoor:** writing original draft, review and editing. **Mridul Khanna:** review and editing, formal analysis. **Amit Sehrawat:** conceptualization, diagnosis and formal analysis. **Kranthi Kumar Jandrasupalli:** investigations and diagnosis, data curation. **Ravi Hari Phulware:** diagnosis, data curation and formal analysis. **Ujjawal Shriwastav:** writing original draft, reviewing editing. All authors: final approval.

## Ethics Statement

Ethics approval was not necessary as per the institutional ethics committee, however, informed consent from the patient was obtained to publish the manuscript.

## Conflicts of Interest

The authors declare no conflicts of interest.

## Data Availability

The data that support the findings of this study are available from the corresponding author upon reasonable request.
